# Blastocyst complementation and interspecies chimeras in gene edited pigs

**DOI:** 10.3389/fcell.2022.1065536

**Published:** 2022-12-08

**Authors:** Yong-ho Choe, Jacob Sorensen, Daniel J. Garry, Mary G. Garry

**Affiliations:** ^1^ Lillehei Heart Institute, University of Minnesota, Minneapolis, MN, United States; ^2^ Cardiovascular Division, Department of Medicine, University of Minnesota, Minneapolis, MN, United States; ^3^ Stem Cell Institute, University of Minnesota, Minneapolis, MN, United States; ^4^ Paul and Sheila Wellstone Muscular Dystrophy Center, University of Minnesota, Minneapolis, MN, United States

**Keywords:** blastocyst complementation, somatic cell nuclear transfer, pigs, ETV2, MyoD, Myf5, MYF6

## Abstract

The only curative therapy for many endstage diseases is allograft organ transplantation. Due to the limited supply of donor organs, relatively few patients are recipients of a transplanted organ. Therefore, new strategies are warranted to address this unmet need. Using gene editing technologies, somatic cell nuclear transfer and human induced pluripotent stem cell technologies, interspecies chimeric organs have been pursued with promising results. In this review, we highlight the overall technical strategy, the successful early results and the hurdles that need to be addressed in order for these approaches to produce a successful organ that could be transplanted in patients with endstage diseases.

## Introduction

Endstage organ failure or acute traumatic injuries are associated with considerable morbidity and mortality. The only curative therapy for many of these terminal or devastating diseases is solid organ transplantation (Garry et al., 2005; Virani et al., 2021). Due to the limited number of organ donors, this curative therapy is available for only a subpopulation of the patients that need these therapies. For example, it is estimated that 200,000 to 300,000 American adults would benefit from orthotopic heart transplantation annually, but only about 3000 adults receive a cardiac transplant (Virani et al., 2021). This disparity is what drives the pursuit of alternative therapies. In addition to endstage organ disease such as heart disease, there are traumatic injuries that threaten limbs and ultimately contribute to volumetric muscle loss (Corona et al., 2015; Greising et al., 2016). Currently, there are limited treatments for volumetric muscle loss and thus lead to considerable morbidity, amputations, lifelong disability and loss of life (Greising et al., 2017). These chronic diseases and traumatic injuries warrant new therapies.

**FIGURE 1:**
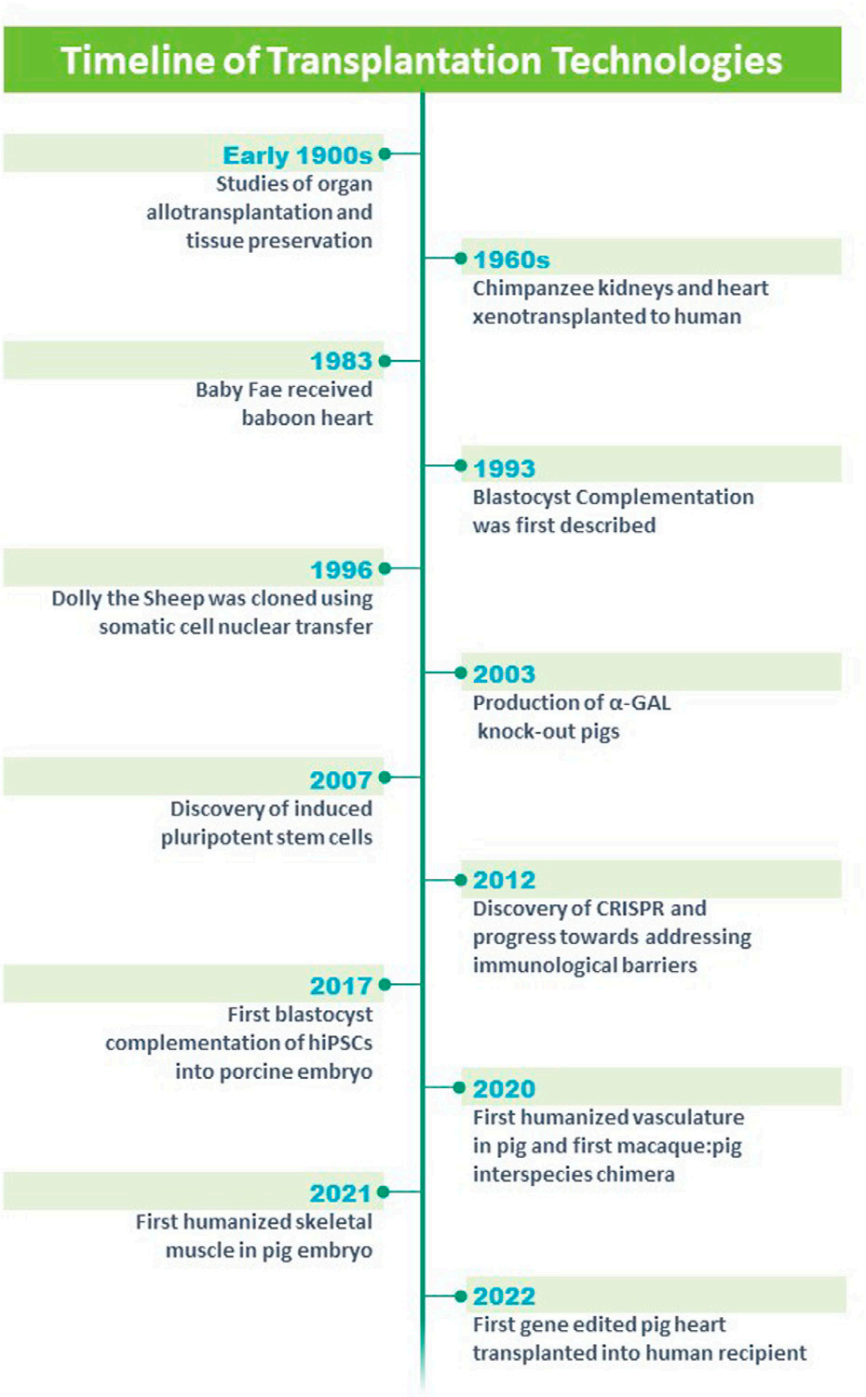
Timeline of technologies and advances in the field of solid organ transplantation. Modified from https://blogs.ubc.ca/xenotransplantationandethics/sample-page/.

Technological advancements such as gene editing (Doudna and Charpentier, 2014; Jinek et al., 2012; Cong et al., 2013) and somatic cell nuclear transfer (SCNT) technologies (Polejaeva et al., 2000; Tian et al., 2003; Galli and Lazzari, 2021) have now allowed for genetic modification of large animals such as the pig (Lai et al., 2002; Yamada et al., 2005; Hisashi et al., 2008; Pierson et al., 2020; Watanabe et al., 2020; Hinrichs et al., 2021; Reichart et al., 2021) (Figure 1). These genetically modified pigs have renewed the interest in xenotransplantation but they also provide a platform for engineering human organs and nonhuman primate cells (interspecies chimeras) in large animals (Wu et al., 2016; Garry and Garry, 2019; Fu et al., 2020; Garry and Garry, 2021). For example, a gene edited pig served as a donor for the first porcine cardiac xenotransplantation which was performed early in 2022 (Graham, 2022; Griffith et al., 2022; Shah and Han, 2022). The recipient of the cardiac xenotransplant survived approximately 2 months and provides a platform for future clinical studies using these gene edited nonhuman organs that would serve as an unlimited organ source for those with terminal diseases (Griffith et al., 2022).

Here, we provide a review of the studies related to cardiac xenotransplantation and blastocyst complementation in the porcine animal model as potential strategies to engineer a suitable organ for transplantation that will have longterm function and decreased requirements for immunosuppressive agents. Additionally, we will highlight the opportunities and the challenges for each of the strategies with the overall goal of engineering organs for research opportunities and clinical treatments.

## Cardiac xenotransplantation: Historical perspective and recent successes

Xenotransplantation has been examined using an array of organs including heart, kidney, vasculature, skin, pancreas, islets and other organs. Alexis Carrel, M.D. was an early pioneer whose research focused on the surgical repair of the vasculature in the early 1900s (Carrel, 1907). Carrel used his knowledge of the vasculature and applied it to the transplant of arteries, veins, kidneys and hearts (heterotopically), limbs and endocrine glands (Carrel and Guthrie, 1905; Carrel, 1912; Carrel, 1914). Further, he focused on tissue preservation and oxygenation of tissues (Carrel, 1907). Collectively, his studies earned him the Nobel Prize in 1912 and these studies provided a platform for allograft transplantation and xenotransplantation (Figure 1).

James D. Hardy undertook the first xenotransplantation using an undersized nonhuman primate heart into a 68 year old male patient who had severe vasculopathy and was supported with mechanical ventilation (Hardy et al., 1964; Cooper, 2012; Cooper et al., 2015) (Figure 1). While the xenotransplanted heart was technically successful, the patient expired in the operating room after 90 min of support, in part, due to the undersized donor organ and rejection.

There was a considerable hiatus for the field of cardiac xenotransplantation until Dr. Leonard Bailey at Loma Linda Hospital undertook a heroic effort by implanting a baboon heart into the newborn Baby Fae who had hypoplastic left heart syndrome (Bailey et al., 1985; Stafford, 2019) (Figure 1). Baby Fae survived for 21 days and ultimately the graft failure was secondary to xenograft rejection (despite the use of immunosuppression agents). These early studies emphasized the importance of the vasculature which was the source of hyperacute rejection and chronic rejection of the xenograft. Emerging technologies began to revolutionize the field. Keith Campbell and Ian Wilmut’s team at the Roslin Institute successfully cloned Dolly who lived from 5 July 1996 to 14 February 2003 and whose birth and lifespan was a milestone for mammalian cloning technology (Campbell et al., 1996; Wilmut et al., 1997; Wilmut et al., 2007) (Figure 1). Further, the advent of gene editing (Jinek et al., 2012; Cong et al., 2013; Doudna and Charpentier, 2014) and reprogramming technologies (using Yamanaka factors (Takahashi and Yamanaka, 2006; Takahashi et al., 2007); provided important platforms for genetically engineering large animal models such as swine (Figure 1). Using these technologies, investigators genetically modified domestic pigs by deleting genes that contributed to hyperacute rejection of the xenotransplanted organ (*α-1,3-galactosyltransferase* or *GGTA1*, *β-1,4-N-acetylgalactosaminyltransferase two* or *B4GalNT2* and *Cytidine monophosphate-N-acetylneuraminic acid hydroxylase* or *CMAH)* and Growth hormone receptor to prevent xenograft overgrowth (Kolber-Simonds et al., 2004; Ahrens et al., 2015; Choi et al., 2017; Sykes and Sachs, 2019; Goerlich et al., 2021; Hinrichs et al., 2021; Reichart et al., 2021) (Figure 1). Further genetic modifications included transgenic overexpression of human proteins to decrease cellular injury (CD47 and Hemoxygenase 1) (Petersen et al., 2010; Ahrens et al., 2015; Cooper et al., 2018), complement activation (hCD55 and hCD46) (Fischer et al., 2016) and coagulopathies (human Endothelial Cell Protein C Receptor and human Thrombomodulin) (Oropeza et al., 2009; Petersen et al., 2009). For the most part (with the exception of the Growth hormone receptor knockout), these genetic modifications targeted the porcine vasculature as these endothelial antigens have been shown to promote hyperacute rejection, delayed xenograft rejection, antibody mediated rejection and chronic xenograft rejection. The hearts from these gene edited pigs, were xenotransplanted into baboons in either a heterotopic position (to assess for rejection) (Lin et al., 1998; Hisashi et al., 2008; Iwase et al., 2015; Goerlich et al., 2020) or in the orthotopic position (to assess for functional performance) (Schmoeckel et al., 1998; Waterworth et al., 1998; Goerlich et al., 2021; Litovsky et al., 2022) (Figure 1). These xenografts performed well and these preclinical studies served as a platform for human studies. On 7 January 2022, a patient with endstage heart failure who was supported by extracorporeal membrane oxygenation (ECMO) received FDA approval for compassionate care use of a cardiac xenotransplantation from a gene edited pig (Figure 1) (Griffith et al., 2022; Pelc and Smiley, 2022; Reardon, 2022). The cardiac xenotransplant surgical procedure was successful although the patient had several complications including aortic dissection, acute kidney injury, thrombocytopenia and infections. The patient survived approximately 2 months, was the longest living survivor of cardiac xenotransplantation and was the world’s first human to receive a porcine heart (Griffith et al., 2022). These efforts have provided an important platform for other strategies aimed at engineering organs for transplantation therapies.

## The use of miniature swine for the production of human organs

Miniature swine have been utilized extensively as biomedical research models of human disease due to their comparable size to humans, docile behavior, and slower growth curves when compared to domestic swine (Table 1). Various breeds including the Hanford (Ho et al., 2010), Sinclair/Hormel/Minnesota Mini (Misfeldt and Grimm, 1994), Yucatan (Panepinto and Phillips, 1986) and Gottingen (Khan et al., 2018) have been bred and each have distinct advantages and uses (Table 2). The use of miniature swine for the production of human organs is equally attractive for the reasons cited above but also for the relatively short gestational times and the ability to use inbred strains such as MGH defined MHC factors (Sykes and Sachs, 2019) and where isolation and breeding techniques can eliminate zoonotic viruses.

**TABLE 1:** Domestic pigs vs. Miniature pigs.

	Domestic pigs	Miniature pigs
**Life span (years)**	6–9	10–15
**Body temperature (°C)**	39.2 ± 0.5	39.2 ± 0.5
**Body Weight (kg)**		
Male	100-300	40-90^a^
Female	100-300	40-90^a^
**Growth rate**	Fast	Slow
**Sexual maturation (months)**	7–9	4–5
**Estrus cycle (days)**		
Frequency	18–24	18–24
Duration of estrus	2–3	2–3
**Gestation period (days)**	112–115	111–114
**Litter size**	8–15	Mean 6, range 3–12
**Breeding period (years)**	4–5	6–8
**Heart rate (bpm)**	60–80	105 ± 7
**Blood pressure (mmHg)**		
Systolic	127 ± 8	128
Diastolic	86 ± 7	80
**MAP**	79–121	68–118
**CO (lpm)**	5–5.3	7–7.6
**Ease of handling**	Poor	Good (small size and docile temperament)
**Genetic background**	Inbred or crossbred	Well characterized
		Inbred or closed herd, outbred

^a^ Variable depending on strains. Table compiled from (McKenzie, 1996; Kahn, 2005; Stricker-Krongrad et al., 2016; Bouchard et al., 2019).

**TABLE 2:** Comparison of various Miniature pig strains.

Parameter	Hanford	Yucatan	Sinclair	Göttingen
**Life span (year)**	10–15	10–15	10–15	10–15
**Body temperature (°C)**	39.2 ± 0.5	39.2 ± 0.5	39.2 ± 0.5	39.2 ± 0.5
**Weight (kg)**				
2 months	8–11	7–9	7–9	3–6
12 months	60–70	55–60	37–42	26–35
24 months	80–95	70–80	55–70	41–53
**Sexual maturation (month)**	4–6	4–6	4–6	3–5
**Estrus cycle (days)**	21	21	21	21
**Litter size (average)**	6.7	6	7.2	6.5
**Heart rate (bpm)**				
Male	106 ± 20.3	91 ± 19.3	133 ± 41	100 ± 7
Female	104 ± 17.3	92.5 ± 12.2	126 ± 43	92 ± 7
**Heart weight (g)** **^a^**				
Male	146.8 ± 27.9	135.9 ± 12.0	63.6 ± 13.2	73.72 ± 5.8
Female	130.8 ± 18.2	121.6 ± 13.3	62.5 ± 10.9	59.76 ± 8.9
**Relative heart weight** **^a^** (average % of body weight)				
Male	0.41	0.5	0.43	0.52
Female	0.4	0.47	0.44	0.41

^a^ Absolute and relative weight of 5–8 month old Hanford, Yucatan, Sinclair miniature pig or 6 month old Göttingen miniature pig. Table compiled from: (Gutierrez et al., 2015; Bouchard et al., 2019; Stricker-Krongrad et al., 2017).

## SCNT and blastocyst complementation to produce exogenic organs

Previous studies have demonstrated that the deletion of genes to produce a mouse embryo that lacks an entire lineage or organ can be completely rescued using blastocyst complementation and pluripotent embryonic stem cells from a different species. For example, genetic mouse studies demonstrated that the deletion of *Pdx1* (the master regulator for the pancreas) resulted in a mouse that lacked a pancreas and died early following birth (Offield et al., 1996). Using blastocyst complementation, the *Pdx1* mutant mouse blastocyst was completely rescued using GFP-labeled rat embryonic stem cells (ESCs) and produced a viable mouse with a GFP-labeled pancreas (reflecting that all the pancreatic cells were derivatives of the GFP-labeled rat ESCs) (Kobayashi et al., 2010). Similarly, blastocyst complementation was undertaken with the *Pdx1* null rat blastocyst and the GFP-labeled mouse ESCs (Yamaguchi et al., 2017). The resulting rat was viable and had a GFP-labeled pancreas. These studies demonstrated the importance of the depleted organ niche, the efficiency and feasibility of blastocyst complementation and that the host embryo determined the growth and the size of complemented organ (i.e. the size of the pancreas which was derived from mouse ESCs was equal to the size of a rat pancreas and not the size of a mouse pancreas). These successes provided the rationale for interspecies complementation using larger animal models.

The first challenge for the field was to define an appropriate large animal model. Previously, baboons, sheep, cows, chimpanzees, pigs and others have served as donors or recipients for xenotransplantation (Crick et al., 1998; Cooper, 2012; Cooper et al., 2015; Sachs, 2017). Each of these large animal models have their strengths and limitations. Increasingly, the pig has been used for these types of studies as they have a number of characteristics including: availability, comparable size to humans, large litter size, relatively short gestational periods and they can be housed within barrier facilities and screened for multiple pathogens. Further characteristics associated with the porcine animal model are highlighted in Table 3.

**TABLE 3:** Characteristics of pigs as a biomedical research model.

		Similarity to humans and advantages
**General physiology**	**Life span**	Compared to other experimental animals (rodents, dogs), the relatively long life span of pigs allows for long-term studies.
	(15–25 years)	
	**Growth rate**	Growing rapidly until reaching adult human size (6 months) than nonhuman species
	**Gestation period** (112–115 days)	The short gestation period and large litter size of pigs: good availability (high productivity, low reproduction costs)
	**Litter size** (8-15)	
	**Organ size and function**	• Similar size, weight and function of pig organs (heart, kidney, skin, GI): benefit for research of surgery training, drug safety, and xenotransplantation
		• Similar metabolism: pigs are widely used for field of toxicology
**Cardiovascular system** (heart, vasculature)		• Similar anatomical size and structure:
		• Heart morphology, size, and weight
		• Structure of atria and ventricles
		• Similar physiological feature
		• Coronary circulation pattern
		• Hemodynamic parameters and vascular remodeling mechanisms
		• Traditional medical material: tissue from porcine hearts has been widely used to develop human heart valve and blood vessel replacements
**Musculoskeletal system**		Similar femoral bone cross-sectional diameter, area, lamellar bone structure, bone regeneration processes, bone mineral density, and concentration
**Pathogen control**		• The well-known species-specific pathogen of pig herd and they have been monitored extensively.
		• Possible to establish pathogen barrier facilities for pig.
**Genome editing**		Advances in genome editing technologies, such as CRISPR-Cas9, somatic cell nuclear transfer (SCNT), and interspecies chimera can now be routinely applied

Table compiled from (Cooper., 2012; Garg et al., 2013; Gutierrez et al., 2015; Renner et al., 2016; Swindle and Smith, 2016; Garry and Garry, 2019; Lunney et al., 2021).

The Nakauchi laboratory generated the first large animal exogenic organ by using blastocyst complementation to deliver allogenic porcine blastomeres to a pancreas disabled porcine (Matsunari et al., 2013). These studies demonstrated the generation of functional pancreata in lineage disabled pigs (Matsurnari et al., 2013). Additionally, the rescue of pigs that were lacking kidneys (Matsunari et al., 2020), livers (Matsunari et al., 2020), vasculature and blood (Das et al., 2020) or pancreas/vasculature/blood (Matsunari et al., 2020) was demonstrated using allogenic blastocyst complementation (Das et al., 2020; Matsunari et al., 2020) or with blastocyst complementation of human cells (Das et al., 2020). These papers provided a platform for the deletion and rescue of the vasculature and blood (either alone or in addition to other lineages) within a chimera which may be essential for the transplantation of an organ without rejection.

The discovery of hiPSCs (Takahashi and Yamanaka, 2006; Takahashi et al., 2007) (Figure 1) facilitated the ability to perform blastocyst complementation using human cells. Accordingly, the Belmonte laboratory delivered various types of hiPSCs in WT porcine parthenotes which were, ultimately, delivered to surrogate animals (Wu et al., 2017). Early stage analysis of these chimeric embryos revealed that the donor stem cells were localized throughout the embryo (using lineage specific antisera) (Wu et al., 2017). The authors concluded that while these technologies were successful, the efficiency of the chimerism was limited.

## Engineering human hematoendothelial lineages using blastocyst complementation

Our laboratories targeted master regulators and pioneer factors that regulated specific lineages and used gene editing technologies to produce organ depleted porcine embryos that would serve as recipients for blastocyst complementation of donor stem cells. Initially, studies focused on the vasculature as this organ has been shown to harbor cell surface receptors/proteins that promote hyperacute rejection, antibody mediated rejection and chronic rejection (Auchincloss and Sachs, 1998; Sykes and Sachs, 2019). Previous studies using independent screens identified ETV2 and global deletion of this gene resulted in nonviable mouse embryos (E8.5-E9.5) that lacked vasculature and blood (Rasmussen et al., 2012; Rasmussen et al., 2013; Shi et al., 2014; Shi et al., 2015; Garry, 2016; Gong et al., 2017; Koyano-Nakagawa and Garry, 2017; Singh et al., 2020). Additional studies, using zebrafish, *xenopus* and mice, defined upstream regulators, downstream regulators and further defined roles in the regulation of cell proliferation, cell migration (Chan et al., 2013; Craig et al., 2015; Singh et al., 2017; Gong et al., 2022) and we have identified ETV2 as a pioneer factor for development of the hematoendothelial lineage (Gong et al., 2022). Furthermore, the genetic deletion of the 3.9 kb upstream fragment of the *Etv2* gene, which harbors the promoter and enhancer modules, phenocopied the global deletion of Etv2 supporting the notion that this fragment contained all the regulatory modules for *Etv2* gene regulation (Koyano-Nakagawa et al., 2015). Deletion of *ETV2* in the pig also resulted in the absence of vasculature and blood and the mutant embryos were nonviable by E16/E17 (Das et al., 2020). Having established an organ (vasculature) depleted porcine model, SCNT and blastocyst complementation using GFP-labeled pig stem cells resulted in the complete rescue of the *ETV2* null phenotype and viable intraspecies chimeric pigs with GFP-labeled vasculature and blood (Das et al., 2020). These intraspecies chimeric studies represent an important accomplishment for interspecies blastocyst complementation. Two GFP-labeled hiPSCs were delivered into the four to six cell *ETV2* null porcine embryo and these interspecies chimeras were transferred to surrogate gilts. Analysis of the chimeric embryos revealed that the hiPSCs rescued the lethal phenotype and differentiated to the endothelial lineage (Figure 1) (Das et al., 2020). Importantly, no hiPSCs or human derivatives were found outside of the hematoendothelial lineages. These studies demonstrated the merit for using an organ depleted strategy (to establish a niche for stem cell homing) in a large animal model. Future studies will be necessary to examine later embryonic development of the interspecies chimeras and the production of a viable human-pig chimera that has a human vasculature.

## Engineering human muscle using blastocyst complementation

**FIGURE 2:**
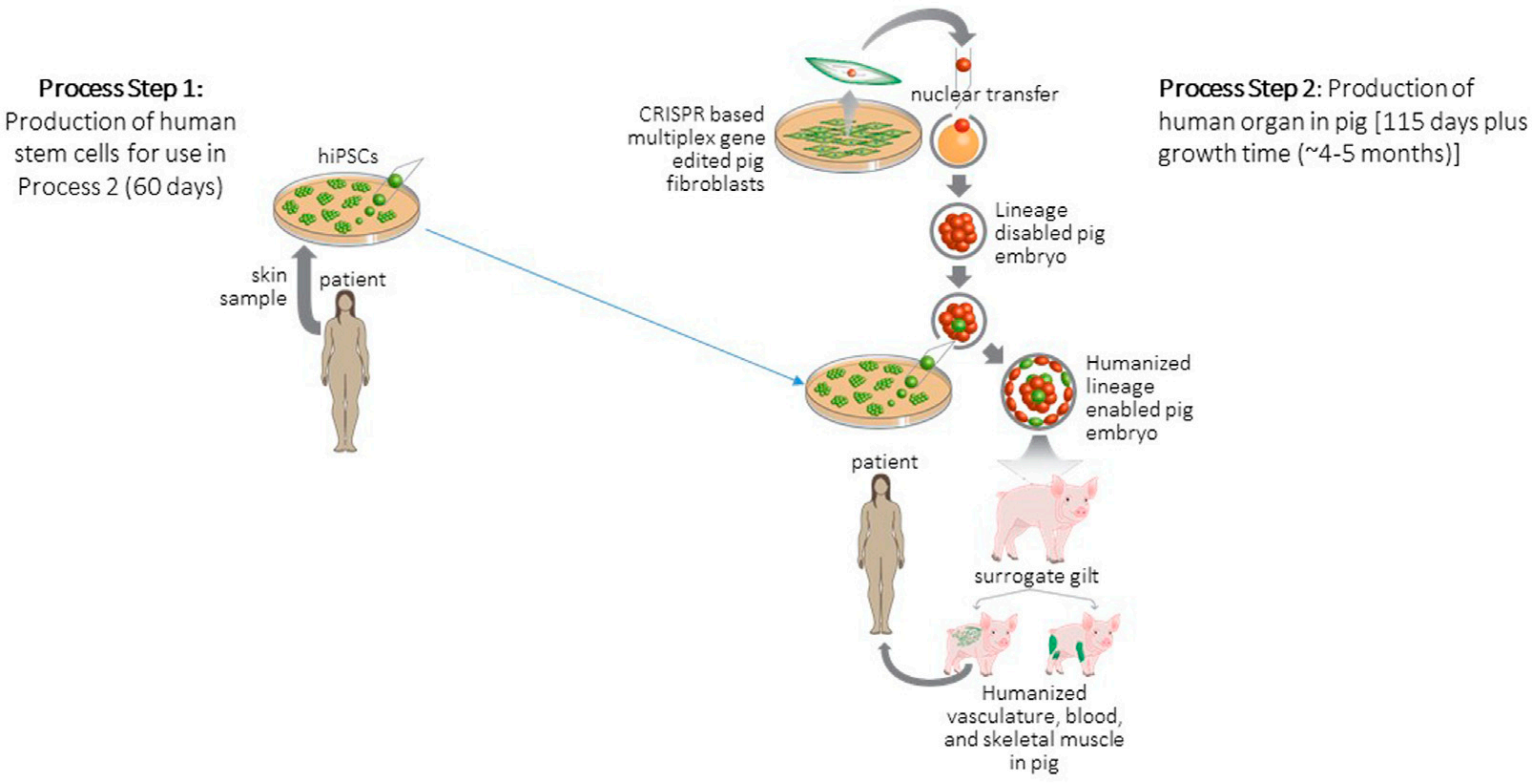
Strategy for generation and transplantation of human organ engineered in pig. Process Step 1: Biopsies are collected from patients to derive hiPSCs. Process Step 2: Porcine embryonic fibroblasts are gene edited to delete a lineage following which these fibroblasts are cloned to produce null porcine embryos. Blastocyst complementation is used to deliver patient derived hiPSCs to null embryos following which the complemented embryos are surgically transferred to a surrogate animal. The offspring of the surrogate develops a human organ to replace the deleted lineage. These human organs are then harvested and transplanted to the patient. Figure modified from (Garry and Garry, 2021).

Previous studies have demonstrated the essential role of the basic helix-loop-helix (bHLH) MYOD family members (MYF5, MYOD, Myogenin and MRF4 also known as MYF6) as master regulators or fundamental determinants for the myogenic lineage (Hasty et al., 1993; Rudnicki et al., 1993; Rawls et al., 1995; Rawls et al., 1998). Elegant studies using the mouse model demonstrated that these myogenic regulatory factors revealed functional redundancy (Rawls et al., 1995; Tajbakhsh et al., 1997; Rawls et al., 1998; Valdez et al., 2000; Kassar-Duchossoy et al., 2004). Using CRISPR/Cas9 gene editing, porcine fibroblasts were genetically modified with *MYF5/MYOD/MYF6* multiplex deletions (Maeng et al., 2021) (Figure 2). These mutant porcine embryos were found to lack myogenic progenitors and the skeletal muscle lineage. This mutant phenotype was completely rescued using blastocyst complementation with GFP-labeled pig stem cells (Maeng et al., 2021). These intraspecies chimeras were viable, ambulatory and indistinguishable from the controls. The skeletal muscle from these intraspecies chimeras was fully developed, labeled with GFP (demonstrating that the myofibers were derivatives of the exogenous GFP-labeled porcine stem cells). The chimeric skeletal muscle had a normal fiber type distribution and satellite cell distribution which were resident within the muscle. Furthermore, the physiological response to stimuli were comparable between controls and the intraspecies chimeras (Maeng et al., 2021). Using blastocyst complementation and GFP-labeled hiPSCs as donor cells, the interspecies chimeras were delivered into surrogate gilts and analyzed at two developmental stages. The analyses of these interspecies chimeras used wholemount immunofluorescence, immunohistochemical, sequence analysis (to confirm the presence of human members of the *MYOD* family and the absence of porcine proteins) and PCR techniques (Maeng et al., 2021). These studies demonstrate that the myotome of the somite was repopulated with human myogenic progenitors that expressed human *MYOD* and human *MYF5*. Furthermore, these human myogenic progenitors migrated to the limbs and formed human skeletal muscle. Importantly, a rigorous analysis was performed and demonstrated that no human stem cells or derivatives were located in the brain or the gametes of the chimeric embryos. Collectively, these studies demonstrated the benefits of establishing a muscle depleted embryo as a recipient for engineering the interspecies chimeras (Maeng et al., 2021).

## Strategies for enhanced efficiencies for the generation of interspecies chimeras

Interspecies chimera production is highly dependent on a number of factors. One factor is the evolutionary distance between the donor cells and the recipient embryo. We know that rat-mouse interspecies chimera formation is relatively efficient and produces viable offspring following blastocyst complementation. The evolutionary distance between rat and mouse species is approximately 25–35 million years (Makalowski and Boguski, 1998). In contrast, the evolutionary distance between humans and mice (rodents) is approximately 95 million years (Makalowski and Boguski, 1998). Likewise, the distance of a common ancestor between humans and pigs is estimated to be more than 90 million years ago (Wei et al., 2018). This divergence reflects the genetic similarity between the respective species. Another factor may be the survival of the donor cells following blastocyst complementation. Studies undertaken in several laboratories have demonstrated the benefit of genetic modification of donor cells to promote cell survival. For example, stem cells that overexpress B-cell lymphoma 2 (*BCL2) and/or MYCN* have shown increased efficiency of chimera production (Labi et al., 2013; Wang et al., 2018; Das et al., 2020; Zhu et al., 2022). These studies demonstrate that programmed cell death related to evolutionary divergent donor cells is an important factor or barrier. While a clear benefit has been demonstrated, chronic overexpression of *BCL2* may be associated with a hypercellular state. Alternatively, strategies have demonstrated that genetic deletion of *TP53* or *Igfr1* in the donor cell population also results in increased efficiency of interspecies chimera production (Maeng et al., 2021). This strategy increased the proliferation of the donor cell population and may facilitate the reprogramming and integration to the recipient embryo. A third strategy has focused on enhancement of donor cell-recipient cell interconnections and communication, metabolic transition, trophectoderm development and cell surface proteins all of which have been shown to impact the efficiency of interspecies chimeras (Zheng et al., 2021). These latter studies have used single cell RNA-seq to define distinct, species-specific molecular programs at various times during early embryogenesis (Gong et al., 2017; Cutter et al., 2019; Das et al., 2020; Lu et al., 2021; Maeng et al., 2021). These studies further demonstrated the species-specific link between the metabolic state and epigenetic and transcriptional gene regulation in the pre-gastrulation embryo and highlighted targets that may be modified in future experiments in order to achieve increased efficiency of primate-porcine chimeras (Theunissen and Jaenisch, 2017; Lu et al., 2021).

## Future studies focused on interspecies chimeras

While initial studies have engineered human-porcine chimeras using an organ-depleted strategy in the porcine recipient, future studies will focus on modifications to enhance the efficiency of interspecies chimeras. Establishment of a developmental niche in the recipient, resulted in successful complementation (Matsunari et al., 2013; Das et al., 2020; Maeng et al., 2021; Nishimura et al., 2021) without the contribution of human stem cells or human derivatives to outside tissues/lineages such as the brain or gametes (Das et al., 2020; Maeng et al., 2021). Future studies using other developmental niche strategies and other human stem cell populations (naïve vs primed vs intermediate hiPSCs) (Wu et al., 2017) will also need to undertake an assessment to examine human cell localization (using immunohistochemistry and PCR) in the embryonic recipient brain and gametes. These quantitative studies will add to the study results that have already been published. Moreover, later stage human-porcine chimeras should be evaluated and characterized using morphological, physiological and molecular (scRNA-seq, ATAC-seq, *etc.*) techniques to assess developmental progression and the comparison of the chimeric organ to the porcine organ. The immunological status and the response of the recipient animal model to the hiPSCs and their derivatives should also be evaluated. The absence of an immunological response by the porcine recipient following blastocyst complementation using GFP-labeled porcine blastomeres supports the notion that tolerance is established in the resulting chimera (Das et al., 2020; Maeng et al., 2021). Future studies will need to dissect the mechanisms associated with the immunological response or tolerance of the recipient animal model to the interspecies (i.e. human-porcine) chimera. The definition of these mechanisms will be an important advance for the field and serve as a platform for studies that will examine the impact of these chimeric organs as a donor supply for patients with chronic and terminal diseases.

## Conclusion

More than 100,000 Americans are awaiting solid organ transplantation and are on the national transplant waiting list. Many more adults could benefit from such curative therapies but due to limited number of donor organs such therapy is not broadly available. Using gene editing technology primarily aimed at the genetic modification of the porcine vasculature, the first porcine cardiac xenotransplant was performed early in 2022. These successes have also paved the path for the generation of human-porcine chimeric organs. Using a gene deletion strategy and blastocyst complementation, human vasculature and human skeletal muscle have been engineered and early chimeric embryos have been examined. Additional studies are warranted that will focus on the developmental progression of the human-porcine chimeric organs, immunological response/tolerance of the recipient animal model, strategies aimed at increased efficiency of interspecies chimeras and the comprehensive characterization of the human chimeras. Collectively, these early studies have generated tremendous enthusiasm and excitement focused on the opportunities to cure endstage diseases and democratize organ transplantation using xenografts and exogenic chimeric organs.
